# Process Design for a Production of Sustainable Materials from Post-Production Clay

**DOI:** 10.3390/ma14040953

**Published:** 2021-02-18

**Authors:** Michał Łach, Reda A. Gado, Joanna Marczyk, Celina Ziejewska, Neslihan Doğan-Sağlamtimur, Janusz Mikuła, Magdalena Szechyńska-Hebda, Marek Hebda

**Affiliations:** 1 Institute of Materials Engineering, Faculty of Material Engineering and Physics, Cracow University of Technology, Warszawska 24, 31-155 Kraków, Poland; michal.lach@pk.edu.pl (M.Ł.); joanna.marczyk@pk.edu.pl (J.M.); celina.ziejewska@pk.edu.pl (C.Z.); jamikula@pk.edu.pl (J.M.); 2 National Research Center (NRC), Department of Refractories, Ceramic and Building Materials, Dokki 12311, Egypt; redagado@gmail.com; 3 Department of Environmental Engineering, Nigde Omer Halisdemir University, 51240 Nigde, Turkey; neslihandogansaglamtimur@gmail.com or; 4 The Franciszek Górski Institute of Plant Physiology Polish Academy of Sciences, Niezapominajek 21, 30-239 Cracow, Poland; 5 The Plant Breeding and Acclimatization Institute—National Research Institute, Radzików, 05-870 Błonie, Poland

**Keywords:** aluminosilicates, alkali activator, calcination, compressive strength

## Abstract

Alkali activated cement (AAC) can be manufactured from industrial by-products to achieve goals of “zero-waste” production. We discuss in detail the AAC production process from (waste) post-production clay, which serves as the calcium-rich material. The effect of different parameters on the changes in properties of the final product, including morphology, phase formation, compressive strength, resistance to the high temperature, and long-term curing is presented. The drying and grinding of clay are required, even if both processes are energy-intensive; the reduction of particle size and the increase of specific surface area is crucial. Furthermore, calcination at 750 °C ensure approximately 20% higher compressive strength of final AAC in comparison to calcination performed at 700 °C. It resulted from the different ratio of phases: Calcite, mullite, quartz, gehlenite, and wollastonite in the final AAC. The type of activators (NaOH, NaOH:KOH mixtures, KOH) affected AAC mechanical properties, significantly. Sodium activators enabled obtaining higher values of strength. However, if KOH is required, the supplementation of initial materials with fly ash or metakaolin could improve the mechanical properties and durability of AAC, even c.a. 28%. The presented results confirm the possibility of recycling post-production clay from the Raciszyn II Jurassic limestone deposit.

## 1. Introduction

Alkali-activated cement (AAC) has been extensively studied in the past years and promoted due to their potential in the development of “green alternatives” to Portland cement. AAC possess reasonable physicomechanical properties, while the environmental benefit of applying AAC technology mainly lies in the reduction of CO_2_ and energy consumption. One of the most important features of the technology behind AAC is that natural materials such as clay and feldspar, as well as industrial by-products such as fly ash (FA) and slag can be used as the initial material [1] for achieving sustainable development goals and “zero-waste” production.

AAC can be generated from a wide range of aluminosilicate precursors, with differing availability and value worldwide. Calcined clays, and especially metakaolin, have become a significant part of the raw materials base of AAC, and can be used both, as a main aluminosilicate precursor of AAC and as a supplementary component in blended activated systems [2]. The most precursor materials used in AAC are structurally disordered, being either glassy (e.g., FA, blast furnace slag) or thermally disrupted layer structures (e.g., metakaolin and other calcined clays) [3,4,5,6]. Therefore, this class of materials is very versatile and locally adaptable [7,8,9]. According to most researchers, the key to their reactivity is average particle size, thus the specific surface area (usually in the range 1.20–38 μm and 2.16–22 m^2^ g^−1^, respectively), and the strain in the bonding network is induced by thermal dihydroxylation [2]. Depending on the raw material components used, AAC can be divided into three main categories:

(i) Cement with high calcium and silicon content [((Na, K)_2_O–CaO–Al_2_O_3_–SiO_2_–H_2_O system); the raw materials such as blast furnace slag or clay (SiO_2_ + CaO > 70%) are alkali-activated, however, the high-pH condition is not required; the first product of the reactions is dominated by a calcium silicate hydrate (C–S–H) and a small percentage of calcium aluminosilicate hydrate (C–A–S–H) gel with a tobermorite-like (mostly Q^2^, and some Q^1^ and Q^3^) structure;
(ii) Cement with low calcium, but higher aluminum and silicon content ((Na,K)_2_O–Al_2_O_3_–SiO_2_–H_2_O system), the raw materials such as metakaolin, granulated blast furnace slags, class F fly ash, are activated by sodium hydroxide (NaOH) or potassium hydroxide (KOH) at a higher pH than that required for the materials with high calcium content, the reactions tend to generate a sodium aluminosilicate hydrate phase (N–A–S–H) with a highly crosslinked (mainly Q^4^) disordered pseudo-zeolitic structure;
(iii) Hybrid cement formed as the result of the alkaline activation of materials with CaO, SiO_2_ and Al_2_O_3_ contents >20% ((Na,K)_2_O–CaO–Al_2_O_3_–SiO_2_–H_2_O]–[(Na,K)_2_O–Al_2_O_3_–SiO_2_–H_2_O), they include materials with a low Portland cement clinker content and over 70% of mineral additions (slag, FA) or blends containing no Portland cement (blast furnace slag, phosphorous slag, and FA); the reaction products are very complex and include C–A–S–H (containing sodium) and (N,C)–A–S–H (high calcium content N–A–S–H) gels [10,11,12].

The reaction, structure, and property of the resulting products are also controlled by several other factors, including the type the alkaline activator, alkali activation time and conditions, curing conditions etc. [2,13].

The durability studies in terms of sulfate attack, acid corrosion, carbonization, or chloride penetration, indicated AAC as materials that poses much better properties than Portland cement (for review see [13]). However, different AAC have shown distinct durability properties due to the different composition being formed, when different raw materials are used. The mechanisms of strength loss, pore structure changes, cracking due to chemical attacks on N–A–S–H in calcium-low AAC, and cracking due to chemical attacks on C–(A)–S–H in calcium-rich AAC are substantially different. The degradation mechanisms of binders and their concretes are more similar for calcium-rich AAC and Portland cements. Incorporation of mineral modifying additions that are single- or multi-component materials is one of the main trends in the development of AAC with enhanced durability. It is also the most effective way to control their economic and ecological performance, as well as the tailoring and design of reaction products assemblage. The mechanism is mainly based on the so-called “filler” effect—an increase of packing density, an improvement of pore structure (shape and size distribution of pores), and a decrease in total porosity, shrinkage, and cracking by forming a crystalline skeleton [2].

Limestone is a low cost and low environmental impact material. Limestone acquired from the Raciszyn II deposit belonging to the WKG Company (Raciszyn, Polska) is a promising raw material for the production of limestone powders and aggregates. The resources of the Upper Jurassic (Oxfordian) limestone are currently estimated at 20 million tons; the area of the Raciszyn II (Raciszyn, Poland) deposit is 31.43 ha and the thickness ranges from 4.1 to 30.7 m. Every year, using the open-cast method and longwall system, approximately 1 million tons of limestone are acquired. The processing of lime aggregate showed that the material usually contains about 60% of fully valuable aggregates of various fractions and about 40% of clay. Waste products generated during the limestone cleaning process serve as calcium-rich material. The first recovered product is pure limestone, the following is lime-quartz sand, which after drying and fractionation is usually utilized as a light filler for mortars and plasters, and the third recovered product is clay. Due to the presence of reactive aluminosilicates, it is an attractive material for the production of AAC [14,15,16,17].

Here, we present the optimization and assessment of the process leading to the generation of AAC from waste post-production calcium-rich clay derived from the Raciszyn II deposit. We discuss in detail the effect of parameters on the cement formation process i.e., thermo-mechanical treatment of post-production clay (drying, grinding, calcination process), calcination temperature, the type of activator used in the polymerization process as well as the changes in properties of the final product, including morphology, phase formation, compressive strength, resistance to aggressive treatment (high annealing temperature), and long-term curing.

## 2. Materials and Methods

The post-production calcium-rich clay was purchased from the WKG Company (Raciszyn, Poland). The chemical composition of the material includes: Ca 38.92 ± 1.64% (CaO 54.5%), O 37.62 ± 0.49%, Si 12.53 ± 0.73% (SiO_2_ 26,8%), Al 6.39 ± 0.42% (Al_2_O_3_ 12.0%), and Fe 2.79 ± 0.12% (Fe_2_O_3_ 4.00%). The dominant phases in the calcium clay mineral composition are calcite and quartz, accompanied by clay minerals from the kaolinite group and calcium silicates [6].

The post-production clay was dried for 48 h at 105 °C and then ground with a laboratory ultra-centrifugal mill (Retsch ZM 200, Retsch, Haan, Germany) using ring sieves with aperture sizes of trapezoid holes 0.12 mm, and 2400 rpm. The calcination (CaCO_3_ -> CaO + CO_2_, endothermic reaction) of post-production clay was carried out at two different temperatures: (i) 700 °C and (ii) 750 °C. The isothermal heating in both cases was performed for 240 min. The calcined clay was cooled to an ambient temperature.

The calcined clay after thermomechanical treatment was mixed with:

(i) Sand (sand composition: SiO_2_ 90.0–90.3%, Fe_2_O_3_ max. 0.2%, TiO_2_ 0.08–0.1%, Al_2_O_3_ 0.4–0.7%, CaO 0.17%, MgO 0.01%; particle size: < 50 µm) and the proportion of clay:sand is 1:1 (wt.%);
(ii) Sand and flay ash (flay ash composition: SiO_2_ 52–58%, Al_2_O_3_ 21–25%, Fe_2_O_3_ 4–7%, K_2_O 3–5%, CaO 2–4%, MgO 2–4%, TiO_2_ 1–2%; received from Skawina Combined Heat and Power Plant, Skawina, Poland) and the proportion of clay:sand:flay ash is 5:4.5:1 (wt.%);
(iii) Sand and metakaolin (metakaolin composition: SiO_2_ 52-56%, Al_2_O_3_ 40–44%, Fe_2_O_3_ 1–2%, K_2_O 1–2%, CaO 0.2–0.6%, TiO_2_ 0.1–0.4%, MgO 0.1–0.4%, received from České lupkové závody, a.s., Nové Strašecí, Czech Republic), the proportion of clay:sand:metakaolin is 4.5:4.5:1 (wt.%).

To produce AAC, the activation process was carried out with: (i) 10 M NaOH and sodium water glass (sodium silicate, SiO_2_/Na_2_O = 2.5) in a ratio of 1:2.5 (wt.%); (ii) a mixture of 10 M NaOH and 10 M KOH, prepared in a ratio of 9:1, 3:1, 1:1 (v%), and the addition of potassium water glass in a ratio of 1:2.5 (wt.%) to each mixture; and (iii) 10 M KOH and potassium water glass (potassium silicate, SiO_2_/K_2_O = 3.0) in a ratio of 1:2.5 (wt.%). The ratio of liquid parts to dry parts was 0.4 by mass. All of the masses, prepared as described above, were formed inside a cube mold with a side length of 150 mm. They were shaken 10 min to fill the entire volume of the mold and to remove air bubbles. The dismantling of the mold took place after 24 h. The samples were cured at an ambient temperature for 14 or 28 days and 3 years.

The morphology of the materials was analyzed with the scanning electron microscopy JEOL-JSM-820 (JEOL, Tokyo, Japan) according to the methodology described in [18]. A water absorption test was carried out following PN-88 B-06250 standard.

The compressive strength test was carried out following EN 12390-3 with the compression machine (Matest, Treviolo, Italy) using a maximum pressure of 3000 kN.

The qualitative determination of mineral composition was carried out with the X-ray powder diffraction (XRD) and Debye–Scherrer method. Diffractograms of all samples were recorded in the scan range from 10° to 80° 2θ using the SmartLab 9.0 X-ray diffractometer (RIGAKU, Tokyo, Japan) and the following parameters: Radiation CuKα, graphite reflective monochromator, lamp voltage of 45 kV, lamp current of 200 mA, step 0.05 2θ, and step counting time 1 s/step. The values of interplanar distances obtained from the diffraction pattern were used to identify the mineral phases of the tested samples, based on data contained in the International Centre for Diffraction Data (ICDD) PDF4+ catalogue and the HighScore Plus computer software (Version: 4.8, Malvern Panalytical B.V., Almelo, The Netherlands).

Spectroscopy analyses were carried out for samples as described earlier [18,19] with an FT-Raman Nicolet NXR 9650 (Thermo Scientific, Waltham, MA, USA) equipped with a Nd:YAG3 + laser with a 1064 nm wavelength (Thermo Scientific, Waltham, MA, USA) on flat geopolymer samples after a month of curing at room temperature. For each material, a minimum of 3 spots was measured. The analyzed surface area of the sample was around 50 μm^2^, while the depth of the focus was smaller than 1 μm. The Raman spectra were recorded in the region of 200–4000 cm^−1^.

The resistance of the final product after 28 days of curing to aggressive conditions was tested by treatment with a temperature of 200 °C, 400 °C, 600 °C, 800 °C, 1000 °C, 1150 °C, and 1200 °C for 120 min.

At least three replicates for each sample type and each treatment type were analyzed. To present variation in data, the standard deviation was calculated and presented on figures and in tables.

## 3. Results and Discussion

### 3.1. Effect of Thermo-Mechanical Treatment

**Figure 1:**
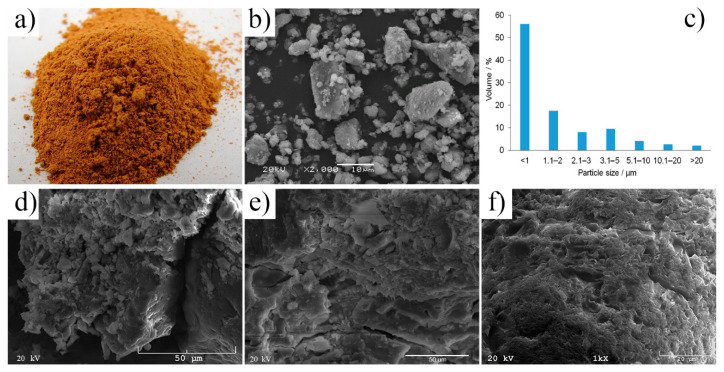
The post-production clay after thermo-mechanical treatment: (**a**) Macroscopic morphology, (**b**) microscopic morphology, (**c**) particle size distribution. The alkali-activated cement formed from post-production clay, mixed with sand, activated with: (**d**) NaOH, (**e**) NaOH and KOH (1:1), and (**f**) KOH; cured for 28 days.

**Figure 2:**
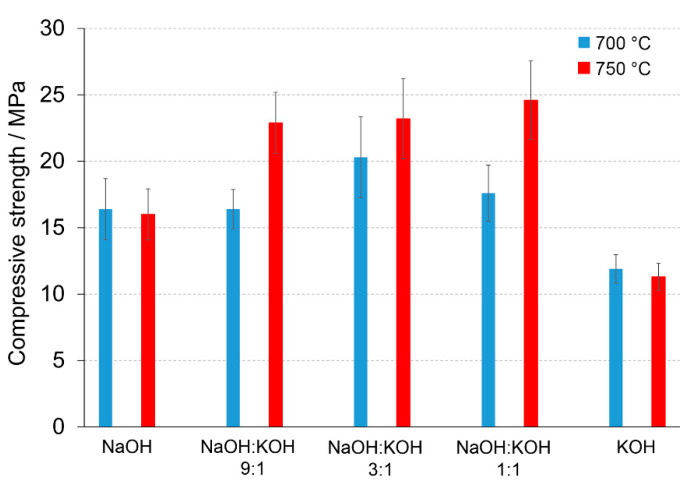
Compressive strength (MPa) of the alkali-activated cement formed from post-production clay mixed with sand; calcined at two different temperatures: 700 °C (blue bars) and 750 °C (red bars); activated by NaOH, NaOH:KOH 9:1, NaOH:KOH 3:1, NaOH:KOH 1:1, and KOH; cured for 28 days.

**Figure 3:**
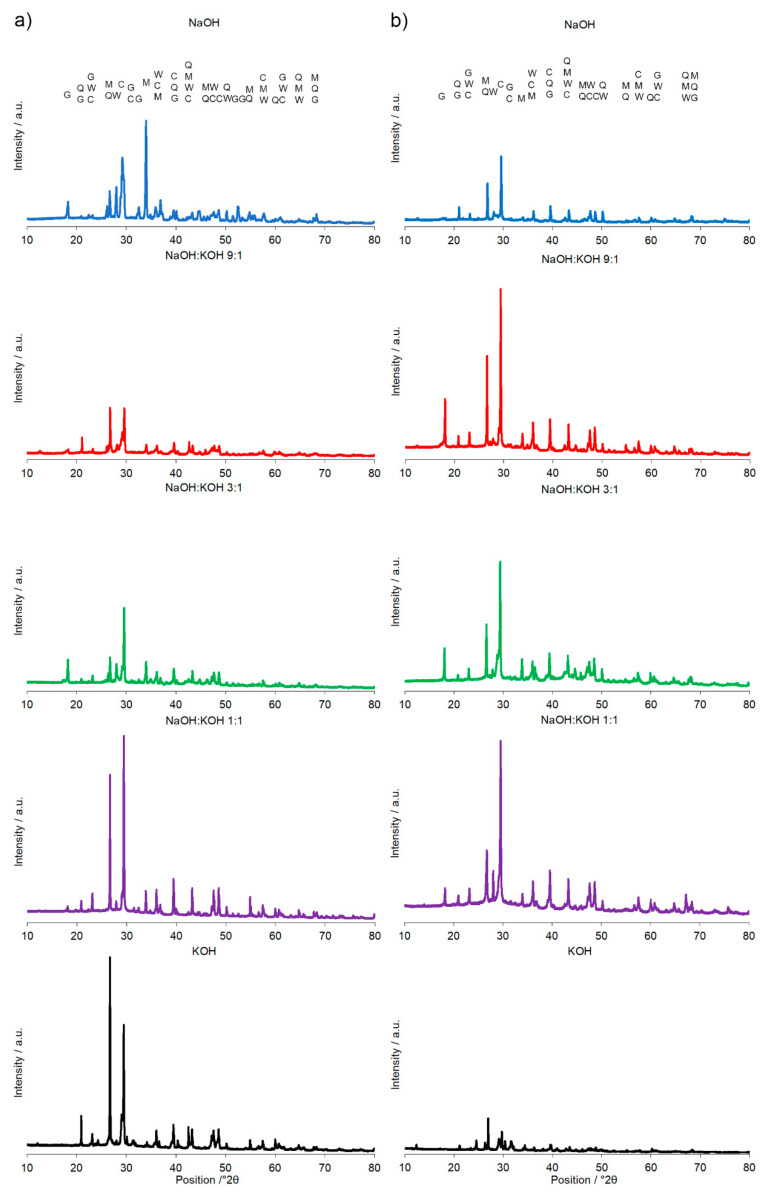
X-ray diffraction pattern of the alkali-activated cement formed from post-production clay mixed with sand; calcined at two different temperatures: 700 °C (**a**) and 750 °C (**b**); activated by NaOH (blue), NaOH:KOH 9:1 (red), NaOH:KOH 3:1 (green), NaOH:KOH 1:1 (purple), and KOH (black); cured for 28 days.

A thermo-mechanical treatment of post-production clay (drying, grinding, and calcination process) had a noticeable effect on the morphology and fineness of clay particles (Figure 1a,b). The clay obtained as post-production material is highly hydrated, lumpy, and compacted, thus unsuitable for direct use in the AAC production process. The analyses showed that the moisture content of the clay in its delivery condition, regardless of the place of its collection, was about 25% [6]. Therefore, the thermo-mechanical treatment of post-production clay i.e., drying and grinding, is required, even if both processes are energy-intensive. The reduction of particle size and the increase of specific surface area are evident after drying and grinding (Figure 1). The final particle size and their distribution in post-production clay were achieved in a range of 0.5–20 µm. They were mostly found in agglomerates with an irregular shape. Once post-production clay was finely ground, it could be recycled as a cementitious material in concrete; the proper size range of particles provided suitable properties of the final product (Figure 1c–e, Figure 2 and Figure 3). A similar conclusion was reached e.g., for clay brick as a pozzolanic material in concrete [20], for a commercial CEM I 42.5N cement [21], or ordinary Portland cement. The compressive strength values of the final product were achieved up to four times higher after fine grinding. Using different mathematical models, high correlations between particle size/specific surface area of the raw material and compressive strength of the produced specimens were established, indicating that particle size have the most noticeable impact on the final product [22]. Regarding alkali activation, the finer particles of the raw material have larger surface areas and react faster with the activating solution; thus more reaction product in finer fractions and unreacted particles in coarser fractions can be found [22,23].

A calcination temperature affected the kinetics and long-term reaction degree between clay particles, thus influenced the performance of final AAC product [24], as it was proved by the comparison of compressive strength of AAC formed after calcination at two different temperatures: 700 °C and 750 °C (Figure 1 and Figure 2). The highest compressive strength was recorded for the AAC samples calcined at 750 °C. In each type of comparative analysis, the compressive strength of AAC was ~20% higher after calcination at 750 °C in comparison to 700 °C. It was found for all of the samples, differently activated and cured (Figure 1 and Figure 2). These results are also confirmed by earlier TG/DTA/MS studies showing that processes occurring during the heating of the post-production clay include: dehydration (220 °C), dihydroxylation (220–700 °C), allotropic conversion of quartz and thermal dissociation of calcite (700–900 °C) [6]. The first process is attributed to evaporation of physically adsorbed water in pores and on the particle surfaces. It is a pre-calcination process; however, endothermal dehydration is important to ensure a proper condition allowing the molecules between the layers of base material break away from the interlayers and loosening the microstructure. At a temperature range of 220–700 °C, the dihydroxylation results in conversion of the crystalline structure of kaolinite [25] to a more disordered material metakaolin with increased pozzolanic activity. The reactivity of calcined clay is strongly related to metakaolin content. Further, the heating up to 700–750 °C maximizes the amount and degree of disorder of metakaolin in the calcined material. The final product is produced by the reactions of dissolution of metakaolin into silicate monomers and aluminate monomers; then polymerization of monomers into aluminosilicate oligomers, and finally fragments combination into larger molecules [18,19]. The absence of specific halo peak with 2θ between 18° and 32° representing the amorphous phase for basic metakaolin in X-ray diffractogram of the final alkali-activated cement (Figure 3), clearly confirmed the high degree of crystallization reactions. Calcite is present in the post-production clay [6], and its decomposition into free lime and carbon dioxide is observed at 600–800 °C [26]. However, the calcite and quartz were still the dominant components at 700 °C [6] and the only observed difference was the lack of kaolinite (dehydroxylated). It was shown that calcite has a negative effect on the specific surface area of calcined material. However, increasing the calcination time to at least 60 min allowed a complete dehydroxylation of kaolinite [26]. Although calcite and quartz were still present at 750 °C, the transformation products, i.e., calcium silicates (Ca_2_SiO_4_ and Ca_2_Si_2_O_7_) were also detected in the samples [6]. Higher calcination temperatures (800–900 °C) could lead to reduced reactivity due to sintering and recrystallization [26]. Generally, at very high-temperature (above 900 °C), clay-based material is fired and SiO_2_ is released according to equation 3(Al_2_O_3_·2SiO_2_) → 3Al_2_O_3_·2SiO_2_ + SiO_2_. In material the glass phase is formed [27]. The interaction of calcite with clay minerals (kaolinite) can also occur, and higher formation of metastable calcium alumino-silicate phases such as wollastonite and gehlenite were reported at higher temperatures [6]. Indeed, more abundant peaks represented wollastonite (50.9°, 25.4°, 29.9°, 36.5° 2θ) and gehlenite (31.3°, 23.9°, 37.4°, 39.2° 2θ) were found for AAC formed from clay mixed with sand and calcined at 750 °C, when compared to samples calcined at 700 °C (Figure 3, Table 1). On the other hand, XRD results showed the sharp peaks characteristic for quartz crystals (at about 26.6°, 20.8°, 50.1°, 59.9° 2θ) and mullite (at about 67.8°, 47.0°, 27.8°, 26.3°, 34.3° 2θ) for samples calcined at 700 °C. This indicates that phases present in base materials were in some part inactive during alkali-activation reaction and stayed as inert phases in AAC. Therefore, one can conclude that the calcination temperature as high as 700 °C is not enough to obtain suitable final products after alkaline activation. The calcination temperature of 750 °C and calcination time of 240 min could be recommended as the most optimal conditions. In the case of these conditions, a significant reduction of calcite and complete dehydroxylation of kaolinite, while higher compressive strength of the final product were reached.

**Figure 4:**
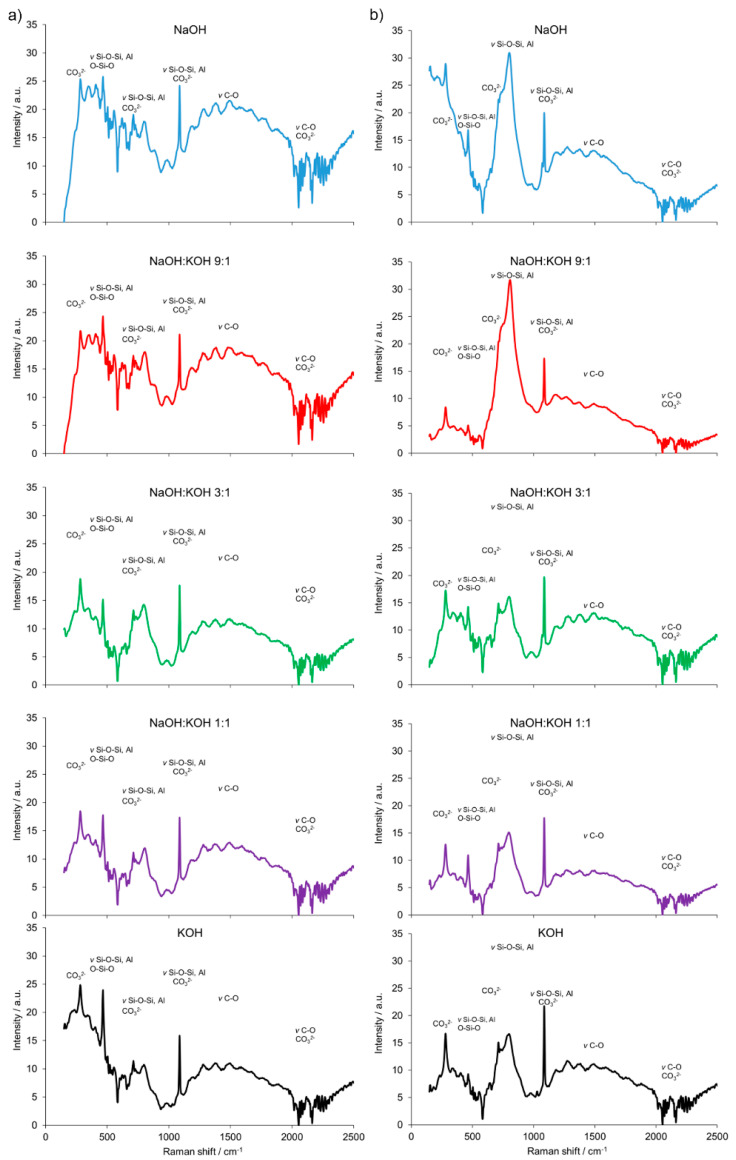
Fourier transform (FT) Raman spectra of the alkali-activated cement formed from post-production clay mixed with sand; calcined at two different temperatures: 700 °C (**a**) and 750 °C (**b**); activated by NaOH (blue), NaOH:KOH 9:1 (red), NaOH:KOH 3:1 (green), NaOH:KOH 1:1 (purple), and KOH (black); cured for 28 days.

Furthermore, the results are consistent with the FT-Raman analysis (Figure 4). The building block of all silicates, amorphous, or crystalline, is a very covalent entity and thus has a well-defined vibrational signature, especially in Raman scattering. The Raman bands attributed to the presence of bridging oxygens associated with tetrahedron polymerization (Si–O–Si, Si–O–Al or O–Si–O) were centered in the region of 500 cm^−1^. Multiple bonding in the regions of 350–500 cm^−1^ and 400–600 cm^−1^ were generally attributed to *ν*_2_-type internal deformations and *ν*_4_-type asymmetric bending vibrations of SiO_4_ tetrahedra, respectively. In the 550–1159 cm^−1^ region, Si–O–Si or Si–O–Al symmetrical stretching vibrations were found. Similarly, a strong complex of bands attributed to the stretching vibration of Si–O groups of the tetrahedral sheets and the absorption bands that belong to Si–O–Al and Si-O-Si bending vibrations, can be found in organoclays with infrared spectroscopy studies [28]. Additionally, a weak and broad Raman band at ca. 885 cm^−1^ appeared for the cement prepared from clay calcined at 700 °C. It was attributed to the symmetric stretching of Si–O–Si, *ν*_1_ of the mullite phase present in the cement. The AlO_4_^−5^ groups were represented by bands at 645 and 482 cm^−1^. They were more abundant in the cement sample prepared from clay calcined at 700 °C. The intense Raman bands associated with CaCO_3_ revealed a heterogeneous concentration of different CaCO_3_ polymorphs in both types of cement (produced from clay calcined at 700 and 750 °C). A small Raman band at 180 cm^−1^ and the intense band ca. 280 cm^−1^ were due to the relative translations between the cationic and anionic groups. The vibration mode *ν*_4_ at 712 cm^−1^ represented symmetric CO_3_ deformation, while the very intensive band at 1085 cm^−1^ corresponded to the vibration mode *ν*_1_ in which all the CO_3_ groups vibrate in identical phases. An intense and narrow Raman band around ca. 283 cm^−1^ was attributed to the presence of calcite. This band could discriminate the types of cement produced from clay calcined at 700 or 750 °C.

### 3.2. Effect of Alkaline Activators

Different activators i.e., NaOH, a mixture of NaOH and KOH, and KOH, affected the structure and mechanical properties of AAC (Figure 1). The microstructure of AAC activated with NaOH was compact, but the particles of the clay were irregular in shape and grouped in the final material in clusters with various sizes. The microstructure was looser (higher total pore number, volume, and size) and cracks were presented, when AAC was produced by the activation with mixture NaOH:KOH. The matrix was compact, a greater number of randomly precipitating individual particles were observed, while the globular clay particles rarely occurred in the reaction product. A smaller total pore number was associated with a larger 1.0–10 µm volume of an individual pore. The different microstructure was developed as a consequence of the reaction between the calcined clay and the KOH solution; the microstructure of AAC had a very uniform, but spongy structure. The developed microstructures resulted in lowering water absorbability along with decreasing the NaOH:KOH ratio (NaOH > NaOH:KOH 9:1 > NaOH:KOH 3:1 > NaOH:KOH 1:1 > KOH, at both types of sample i.e., calcined at 700 and 750 °C, Table 2).

A significant influence of the activator on the compressive strength of AAC formed from post-production clay mixed with sand and calcined at two different temperatures was observed (Figure 2, Figure S1 in Supplementary Materials). The use of the NaOH solution or mixture NaOH with KOH did not differentiate the values of compressive strength of AAC prepared from clay calcined at 700 °C. In contrast, the activator system significantly changed the values of the compressive strength, when clay was calcined at 750 °C. In NaOH-activated AAC, unreacted clay particles interrupted the continuity of the matrix and caused low compressive strength. In contrast, the superficial continuity could develop mechanical consistency, as the highest compressive strength was found for samples activated with the NaOH:KOH mixture. The effect of KOH activation was the worst, in both cases, AAC from clay calcined at 700 and 750 °C. The compressive strength of AAC developed already after a curing period of 14 days and did not changed after 28 days (providing that the same type of activator and calcination temperature, Figure S1 in Supplementary Materials). The XRD (Figure 2, Table 1) showed that the above-described results are consistent with the mineralogical and chemical characteristics of AAC. A higher content of quartz and mullite remaining from base materials, lower content of calcite, and newly produced phases i.e., wollastonite, gehlenite were found and resulted in a lower compressive strength of KOH-activated samples. The peak at about 26.6° 2θ representing quartz and mullite as well as the peak at about 29.4° 2θ representing calcite discriminated very well the AAC that were treated with different activators. The application of NaOH, NaOH:KOH 9:1, and NaOH:KOH 3:1 after clay calcination at a lower temperature resulted in low intensities of both peaks, while their intensities increased along with the increase in KOH concentration (NaOH:KOH 1:1, KOH). In contrast, applying the NaOH:KOH mixture for clay calcined at a higher temperature caused the improvement of calcite along with a higher NaOH concentration (NaOH:KOH 9:1 > NaOH:KOH 3:1 > NaOH:KOH 1:1). Similarly, the Raman bands attributed to the tetrahedron polymerization centered in the region below 500 cm^−1^ and between 600 and 1000 cm^−1^ lowered with the decreasing NaOH:KOH ratio. Concluding, the raw material seemed to be active depending on the combined condition of the calcination vs activation processes. Sodium activators enabled obtaining higher values of strength while potassium activators guaranteed dimensional stability as well as a lack of cracking.

### 3.3. Effect of Chemical Composition

**Figure 5:**
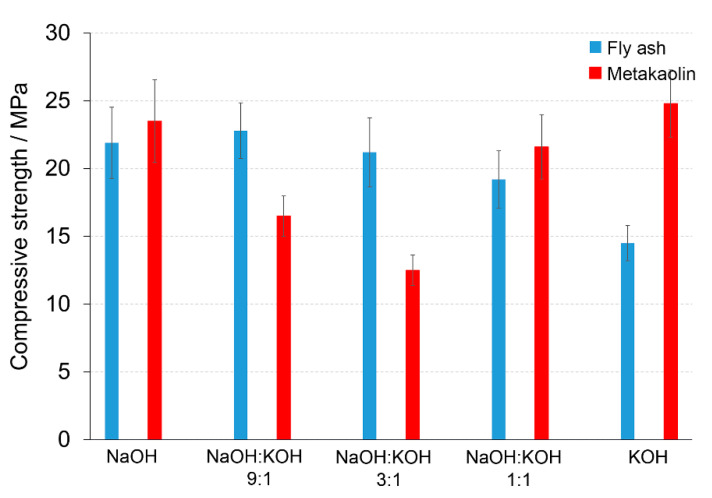
Compressive strength of the alkali-activated cement formed from post-production clay mixed with sand and additives: Fly ash (blue bars), metakaolin (red bars); calcined at 750 °C; activated by NaOH, NaOH:KOH 9:1, NaOH:KOH 3:1, NaOH:KOH 1:1, and KOH; cured for 28 days.

The dominant phases of post-production clay contained Ca 38.92 ± 1.64% (CaO 54.5 %), Si 12.53 ± 0.73% (SiO_2_ 26.8%), and Al 6.39 ± 0.42% (Al_2_O_3_ 12.0%). The chemical composition of final AAC was modified by mixing the post-production clay with: (i) Sand (Ca 0.12, Si 42.09, Al 0.15) to obtain Ca/Si = 0.71 and Si/Al = 8.36; (ii) sand and FA (Ca 2.14, Si 26.13, Al 6.09) to obtain Ca/Si = 0.65 and Si/Al = 7.60; and (iii) sand and metakaolin (Ca 0.29, Si 25.20, Al 11.12) to obtain Ca/Si = 0.64 and Si/Al = 6.57. The additives affected the compressive strength of the produced materials in the mode depending on the used activators. The FA addition (AAC + FA) changed the compressive strength significantly, provided that NaOH or KOH were used as an activator. After activation with NaOH, AAC + FA (Figure 5) increased the compressive strength c.a. 70 or 37%, when the samples were cured 14 or 28 days, respectively, in comparison to AAC (Figure 2, Figure S1 in Supplementary Materials). The compressive strength improved c.a. 28%, when AAC + FA activated with KOH, independently on the curing time. The FA addition did not change compressive strength when NaOH:KOH mixtures were used. The activators with a changing NaOH:KOH ratio influenced the compressive strength of the AAC with the addition of metakaolin (AAC + M) (Figure 5) in the opposite way than AAC (Figure 2). The highest values of the compressive strength were received due to the activation of AAC + M with NaOH or KOH. The NaOH:KOH mixtures reduced the compressive strength, particularly when AAC + M was activated with NaOH:KOH, applied in the proportion of 9:1 and 3:1. In the case of AAC + M, there was no significant reduction in the compressive strength between 14 and 28 days of curing (Figure S1 in Supplementary Materials). The beneficial effect of metakaolin addition towards the improvement of the alkali activation potential was also explored and developed as an integrated approach that will result in their valorization and the production of high added value products that can be used as binders or construction elements, in line with the zero-waste approach and circular economy principles [29].

**Figure 6:**
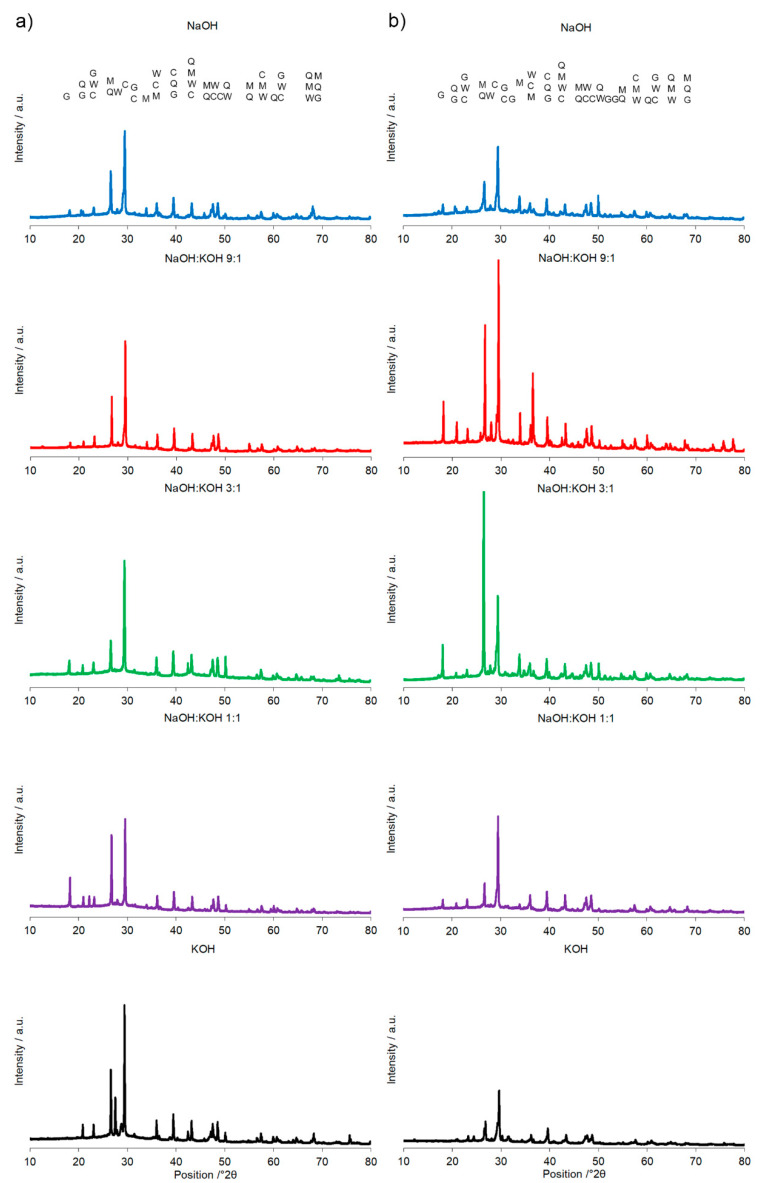
X-ray diffraction pattern of the alkali-activated cement formed from post-production clay mixed with sand and additives: (**a**) Fly ash, (**b**) metakaolin; calcined at 750 °C; activated by NaOH (blue bars), NaOH:KOH 9:1 (red bars), NaOH:KOH 3:1 (green bars), NaOH:KOH 1:1 (purple bars), and KOH (black bars); cured for 28 days.

The most specific alterations, recorded during XRD analysis (Figure 6, Table 3) that can be correlated with the pattern of the changes in the compressive strength, were sharp peaks of quartz/mullite at about 26.6° 2θ vs the peak representing calcite at about 29.4° 2θ. In comparison to AAC, the increase in the intensity of peak representing calcite, but not quartz and mullite, was found for activation of AAC + FA with NaOH and KOH. It indicated a more effective process of cement formation and allowed the development of higher compressive strength. In the same way, higher intensity of peaks representing quartz and mullite and lower intensity of peaks representing calcite, was recorded for AAC + M activated with the NaOH:KOH mixture that was applied in the proportion of 9:1 and 3:1. In these activation systems, quartz and mullite as the remaining phases of the initial materials that lowered the compressive strength of such samples [30]. Earlier studies confirmed that quartz does not participate and completely remains in the structure as fine particles [31], and thus in most cases is considered an undesirable impurity in clay minerals. A high hardness of quartz particles can retard crack growth in the cement matrix. In contrast, mullite as dispersed elongated crystal remaining from initial materials or dissolved during the NaOH treatment and solidified partly into Na-Al-silicates revealed a very strong interfacial bonding between the crystals and cement matrix. Their extensive interaction with the cement converted the strong, flexible, and tough crystals into fragile, brittle ones. As results, the cracks generated in the AAC propagated through the crystals’ ruptures [32]. The wollastonite micro-fibers can delay cracking time by reducing capillary stresses, drying shrinkage, permeability, and leaching [33]. In KOH-treated AAC, higher wollastonite content was observed (Figure 4). KOH developed a microstructure and lower absorbability (Figure 1, Table 2), in agreement with this statement. However, wollastonite as an inert mineral does not contribute to strength development, unless its content exceeds 10–20% [33,34]. At a higher content, the role of wollastonite in bridging the micro-cracks can be expressed and it led to a delay in micro-crack coalescence and higher compressive strength.

**Figure 7:**
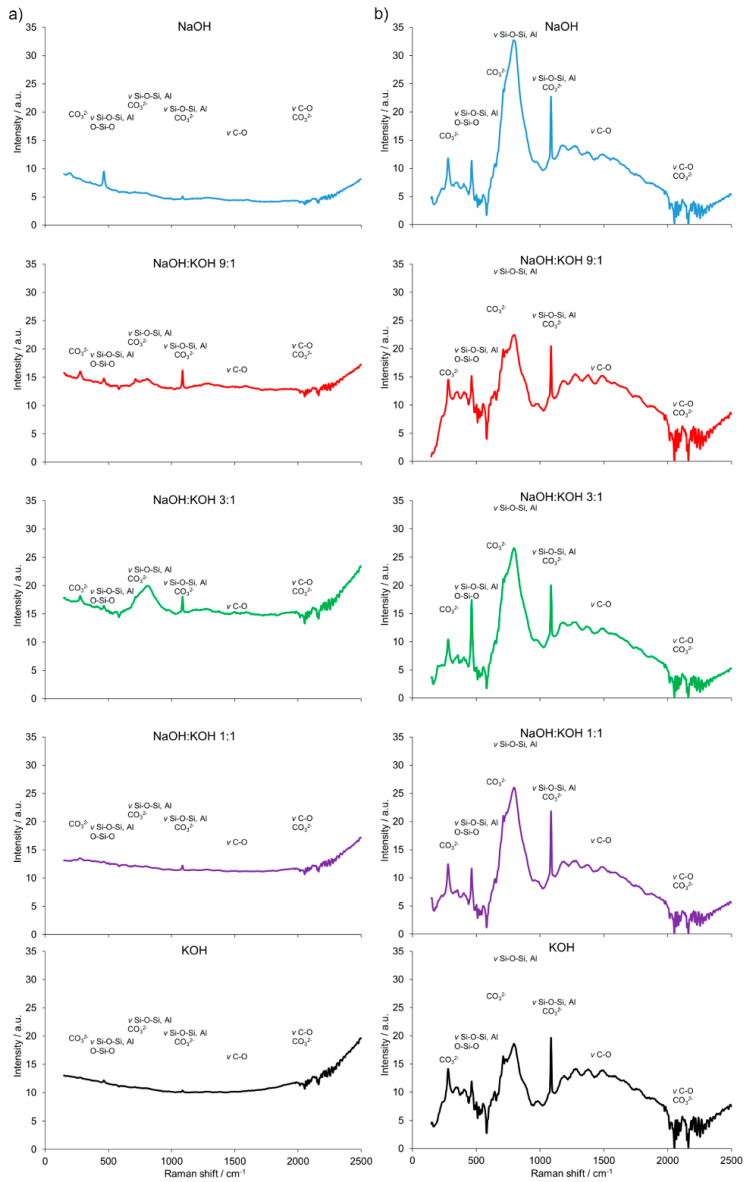
Fourier transform (FT) Raman spectra of the alkali-activated cement formed from post-production clay mixed with sand and additives: Fly ash (**a**), metakaolin (**b**); calcined at 750 °C; activated by NaOH (blue bars), NaOH:KOH 9:1 (red bars), NaOH:KOH 3:1 (green bars), NaOH:KOH 1:1 (purple bars), and KOH (black bars); cured for 28 days.

The results of the FT-Raman shift indicated significant differences in the AAC + FA (Figure 7a) pattern when compared to AAC (Figure 4b) and AAC + M (Figure 7b). The presence of bridging oxygen (Si–O–Si, Si–O–Al or O–Si–O) associated with tetrahedron polymerization was confirmed by bands centered in the region of 350–600 cm^−1^. Similarly, most of the bands related to Si–O–Si, AlO_4_^−5_^ groups, and CO_3_ deformation were presented. However, the spectra of AAC + FA were in most cases flattened and wide. Reduced intensity relative to the shifts characteristic for the Si-O tetrahedral stretch rises is observed along with the loss of structural order. Therefore, the Raman spectra suggest the presence of more amorphous than crystalline phases, primarily because of the absence or presence of spatial order and long-range translational symmetry, respectively [35]. This effect followed the decreasing NaOH to KOH ratio of the used activators. In contrast, spectra from AAC + M were similar to AAC, with relatively higher intensities of bands that are characteristic for Si–O–Si, Si–O–Al, or O–Si–O in the region <500 cm^−1^.

KOH was considered above as an unsuitable activator, due to very low values of compressive strength received for the AAC prepared with sand. However, if KOH is required, the supplementation of base materials with 10% FA or 10% M could improve mechanical properties, slightly or significantly, respectively. KOH provides a greater extent of dissolution due to its higher level of alkalinity. However, if the mixture exceeded its saturation point, the KOH solution has been shown to decrease strength and Si/Al leaching of KOH-based cement occurred more often than those derived from NaOH activation. Altogether, a combination of KOH and NaOH can act in a synergistic way to promote the product of optimal chemical and mechanical characteristics, provided a proper composition of raw materials.

### 3.4. Durability of AAC under Suboptimal Conditions

Table 4 presents the results of the temperature on the compressive strength of AAC. The compressive strength gradually decreased along with the temperature increase from 20 to 600 °C, then upgraded at 800 to 1000 °C, and finally was reduced at 1200 °C. A significant strength loss after heating the samples at 200–600 °C was related to the appearance of fractures in the structure of the material. They result from the rapid evaporation of the chemically-bonded water inside the pores [36], the decomposition of calcite [30], and incompatibility between the shrinking matrix and expanding quartz. As the temperature elevated from 800 to 1000 °C, the phase transition of matrix products into crystal phases e.g., β–wollastonite and gehlenite can contribute to the further deterioration of AAC [37]. Therefore, it is worth noticing that lower changes in the compressive strength during heating were found for KOH-activated AAC, probably due to the higher volume stability and fewer surface cracks in comparison to NaOH-activated AAC. Additionally, the higher content of wollastonite and gehlenite already in untreated samples could promote the process of filling the cracks with the molten phases and bonding the solid particles together resulted in the increase of matrix density and strength. During the annealing of the samples above 1000 °C, a glass-like structure appeared on the surface of the material, and pore pressure grew gradually as a result of heat transfer and gas evaporation. When the vapor pressure exceeds the limit of the matrix, intensive thermal cracking and spalling, the pore structure collapsing, and further contraction could be observed [38].

### 3.5. Long-Term Durability of AAC

As presented above, the strength of the AAC is dependent on a proper choice of an alkaline activator and initial material composition. These results were described for samples curing 28 days. During long-term curing, the carbonation process can result in a more porous binder matrix, leading to strength loss. The carbonation of AAC can cause a loss of cohesion in the matrix and significantly improves the porosity, increasing further carbonation. This weakened the silicate binding and production of coarser porosity are due to the decalcification of C–(A)–S–H. Many different mixes showed extensive microcracking of the binder matrix. However the AAC, developed within this study, were substantially less affected. The improvement in compressive strength was observed in the case of each treatment and activation system (Table 5). The most considerable improvement was found in the case of AAC calcined at 700 °C and KOH-activated samples (in comparison to 28 days of curing, Figure 2). The significant development of compressive strength was also observed when the samples with additives were tested. The exception was AAC + M activated with KOH (only a slight progression in compressive strength values between 28 days and 3 years was observed). The AAC has a rather slow strength gain with time however, the strength can increase by even 2–2.6 times after two years [39]. The authors explained that the gain in strength with time was caused by additional reaction products generated during the reaction between alkali activator and microspheres [40]. The strength increase probably relates to the transformation of aluminosilicate gel to more stable structures.

## 4. Conclusions

As a result of research, a novel recycling procedure was proposed that utilize the post-production clay from the Raciszyn II Jurassic limestone deposit. This industrial by-product can be used as the initial material for the production of alkaline-activated cement with good mechanical properties. In Poland, despite a large amount of this type of waste, so far a suitable solution has not been found to achieve “zero-waste” production. In the short-term and long-term scale, the mechanical properties, including the compressive strength of the produced AAC, depended mainly on: (i) The process of preparing the raw material, (ii) the type of activator used, and (iii) the additives. It was demonstrated that in order to obtain the best possible strength properties of AAC produced from post-production clay, the thermo-mechanical treatment of clay i.e., drying and grinding are required, even if both processes are energy-intensive. The reduction of particle size and increase of specific surface area are key factors influencing the reactivity of the base material. Furthermore, the raw material seemed to be active dependently on the combined condition of calcination vs activation processes. The calcination temperature of 750 °C and calcination time of 240 min could be recommended as the most optimal conditions. In the case of activators, it has been shown that the type of activator and their ratio has a large impact on the quality of the final products obtained. Innovation is the use of a combination of two types of alkaline activators: Sodium and potassium in different proportions. Usually, these activators are used individually for alkaline activation, not in combination. The sodium activators enabled obtaining higher values of strength, while potassium activators guaranteed dimensional stability as well as a lack of cracking. Considering the compressive strength, the best results, however, were obtained for activation with individually applied NaOH. The supplementation of base materials with 10% fly ash or 10% metakaolin could significantly improve the mechanical properties of ACC. After activation with NaOH, AAC + FA increased its compressive strength c.a. 70%. Although KOH was considered a less suitable activator for the AAC prepared with sand, the compressive strength also improved c.a. 28%, when fly ash or metakaolin was added to KOH-activated AAC. The results described are the base of a patent application (the Patent Office of the Republic of Poland). Some limitations in order to use the procedure may result from activator costs. The potassium activator costs are much higher than the sodium one, which is an important factor for large-scale production.

## Figures and Tables

**Table 1:** Phases (%) of the alkali-activated cement formed from post-production clay mixed with sand; calcined at two different temperatures: 700 °C and 750 °C; activated by NaOH, NaOH:KOH 9:1, NaOH:KOH 3:1, NaOH:KOH 1:1, and KOH; cured for 28 days.

Phases	Temperature	NaOH	NaOH:KOH 9:1	NaOH:KOH 3:1	NaOH:KOH 1:1	KOH
Quartz (SiO_2_)	700 °C	14.3	20.1	22.2	23.9	35.1
	750 °C	16.5	19.5	13.5	19.4	6.3
Mullite (Al_6_Si_2_O_13_)	700 °C	-	-	0.6	0.9	2.8
	750 °C	-	-	0.2	0.5	3.0
Calcite (CaCO_3_)	700 °C	85.7	77.4	77.2	70.6	56.1
	750 °C	82.2	74.4	74.5	66.4	36.8
Wollastonite (CaSiO_3_)	700 °C	-	2.5	-	3.8	4.5
	750 °C	1.3	5.6	10.9	12.5	25.9
Gehlenite (Ca_2_Al_2_SiO_7_)	700 °C	-	-	-	0.8	1.5
	750 °C	-	0.5	0.9	1.2	28.0

**Table 2:** Absorbability (%) of the alkali-activated cement formed from post-production clay mixed with sand; calcined at two different temperatures: 700 °C and 750 °C; activated by NaOH, NaOH:KOH 9:1, NaOH:KOH 3:1, NaOH:KOH 1:1, and KOH; cured for 28 days.

Temp	NaOH	NaOH:KOH 9:1	NaOH:KOH 3:1	NaOH:KOH 1:1	KOH
700 °C	2.60 ± 0.19	2.18 ± 0.16	2.01 ± 0.14	2.45 ± 0.21	1.43 ± 0.11
750 °C	3.65 ± 0.47	3.37 ± 0.28	4.22 ± 0.35	3.13 ± 0.24	2.10 ± 0.17

**Table 3:** Phases of the alkali-activated cement formed from post-production clay mixed with sand and additives: Fly ash (FA), metakaolin (M); calcined at 750 °C; activated by NaOH, NaOH:KOH 9:1, NaOH:KOH 3:1, NaOH:KOH 1:1, and KOH; cured for 28 days.

Phases	Additives	NaOH	NaOH:KOH 9:1	NaOH:KOH 3:1	NaOH:KOH 1:1	KOH
Quartz (SiO_2_)	FA	19.1	21.2	18.9	25.5	17.3
	M	24.5	25.2	28.1	24.6	24.5
Mullite (Al_6_Si_2_O_13_)	FA	16.2	17.1	15.6	23.4	17.6
	M	15.5	16.1	18.5	15.6	15.2
Calcite (CaCO_3_)	FA	60.6	58.1	60.3	50.3	61.3
	M	55.3	57.8	53.2	58.3	55.7
Wollastonite (CaSiO_3_)	FA	2.7	2.3	3.1	1.7	3.7
	M	2.8	0.5	0.1	0.9	3.1
Gehlenite (Ca_2_Al_2_SiO_7_)	FA	1.4	1.3	2.1	1.1	0.1
	M	1.9	0.4	0.1	0.6	1.5

**Table 4:** Effect of the annealing temperature (200–1200 °C) on compressive strength of the alkali-activated cement formed from post-production clay mixed with sand; calcined at 750 °C; activated by NaOH, NaOH:KOH 9:1, NaOH:KOH 3:1, NaOH:KOH 1:1, and KOH; cured for 28 days.

Temperature	NaOH	NaOH:KOH 9:1	NaOH:KOH 3:1	NaOH:KOH 1:1	KOH
200 °C	9.1 ± 1.18	8.0 ± 0.88	8.4 ± 1.01	8.4 ± 1.09	12.9 ± 1.94
400 °C	6.4 ± 0.58	6.9 ± 0.55	7.0 ± 0.49	7.2 ± 0.51	9.8 ± 1.27
600 °C	4.8 ± 0.38	4.7 ± 0.38	6.5 ± 0.46	4.9 ± 0.39	7.4 ± 0.67
800 °C	9.8 ± 1.27	9.5 ± 1.33	10.0 ± 1.39	10.2 ± 1.33	7.7 ± 0.69
1000 °C	22.6 ± 3.39	24.7 ± 4.20	21.2 ± 3.39	19.5 ± 2.73	23.5 ± 3.76
1150 °C	23.5 ± 3.37	30.8 ± 5.24	29.4 ± 5.02	31.2 ± 5.39	24.7 ± 3.72
1200 °C	14.2 ± 1.99	15.1 ± 2.27	21.7 ± 3.47	22.2 ± 3.54	21.0 ± 3.17

**Table 5:** Compressive strength of the alkali-activated cement formed from post-production clay mixed with sand (AAC), sand and fly ash (AAC + FA), sand and metakaolin (AAC+M); calcined at two different temperatures: 700 and 750 °C; activated by NaOH, NaOH:KOH 9:1, NaOH:KOH 3:1, NaOH:KOH 1:1, and KOH; cured for 3 years.

Sample/ Calcination Temperature	NaOH	NaOH:KOH 9:1	NaOH:KOH 3:1	NaOH:KOH 1:1	KOH
AAC/700 °C	30.41 ± 3.54	28.88 ± 4.32	28.99 ± 3.32	34.47 ± 4.54	28.70 ± 3.25
AAC/750 °C	22.13 ± 2.38	18.57 ± 1.65	29.70 ± 4.12	28.74 ± 3.28	27.30 ± 3.84
AAC + FA/750 °C	40.56 ± 4.77	33.40 ± 3.02	49.32 ± 6.23	31.83 ± 3.81	43.10 ± 5.13
AAC + M/750 °C	48.10 ± 7.56	45.91 ± 6.78	45.46 ± 7.10	47.02 ± 6.34	29.00 ± 2.97

## Data Availability

No new data were created or analyzed in this study. Data sharing is not applicable to this article.

## Supplementary Materials

The following are available online at https://www.mdpi.com/1996-1944/14/4/953/s1, Figure S1: Compressive strength of the alkali-activated cement formed from post-production clay mixed with sand; calcined at two different temperatures: (a) 700 °C and (b) 750 °C; or formed from post-production clay mixed with sand and additives: (c) fly ash, (d) metakaolin; calcined at 750 °C; activated by NaOH (blue bars), NaOH:KOH 9:1 (red bars), NaOH:KOH 3:1 (green bars), NaOH:KOH 1:1 (purple bars), and KOH (black bars); cured for 14 and 28 days. Figure S2: X-ray diffraction pattern of the alkali-activated cement formed from post-production clay mixed with sand; calcined at two different temperatures: (a) 700 °C and (b) 750 °C; or formed from post-production clay mixed with sand and additives: (c) fly ash, (d) metakaolin; calcined at 750 °C; activated by NaOH (blue), NaOH:KOH 9:1 (red), NaOH:KOH 3:1 (green), NaOH:KOH 1:1 (purple), and KOH (black); cured for 28 days.
