# Supplementary material for: Process Design for a Production of Sustainable Materials from Post-Production Clay
Source: Materials (Basel). 2021 Feb 18;14(4):953. doi: 10.3390/ma14040953 (PMC7922404; doi:10.3390/ma14040953)
Supplement: Supplementary file 1 [file materials-14-00953-s001.pdf]

Article

Process design for a production of sustainable materials from post-production clay

Michał Łach[1], Reda A. Gado[2], Joanna Marczyk[1], Celina Ziejewska[1], Neslihan Doğan-Sağlamtimur[3], Janusz Mikuła[1], Magdalena Szechyńska-Hebda[4,5*] and Marek Hebda[1*]

[1] Institute of Materials Engineering, Faculty of Material Engineering and Physics, Cracow University of Technology, Warszawska 24, 31-155 Kraków, Poland

[2] Department of Refractories, Ceramic and Building Materials, National Research Center (NRC), 12311 Dokki, Egypt

[3] Department of Environmental Engineering, Nigde Omer Halisdemir University, 51240 Nigde, Turkey

[4] The *Franciszek Górski* Institute of Plant Physiology Polish Academy of Sciences, Niezapominajek 21, 30-239, Cracow, Poland

[5] The Plant Breeding and Acclimatization Institute - National Research Institute, Radzików, 05-870 Błonie, Poland

* Corresponding author. e-mail address: mhebda@pk.edu.pl tel.: +48 12628342, e-mail address: m.szechynska@ifr-pan.edu.pl tel.: +48 12 4251833

Supplementary Materials: Figure S1 and Figure S2

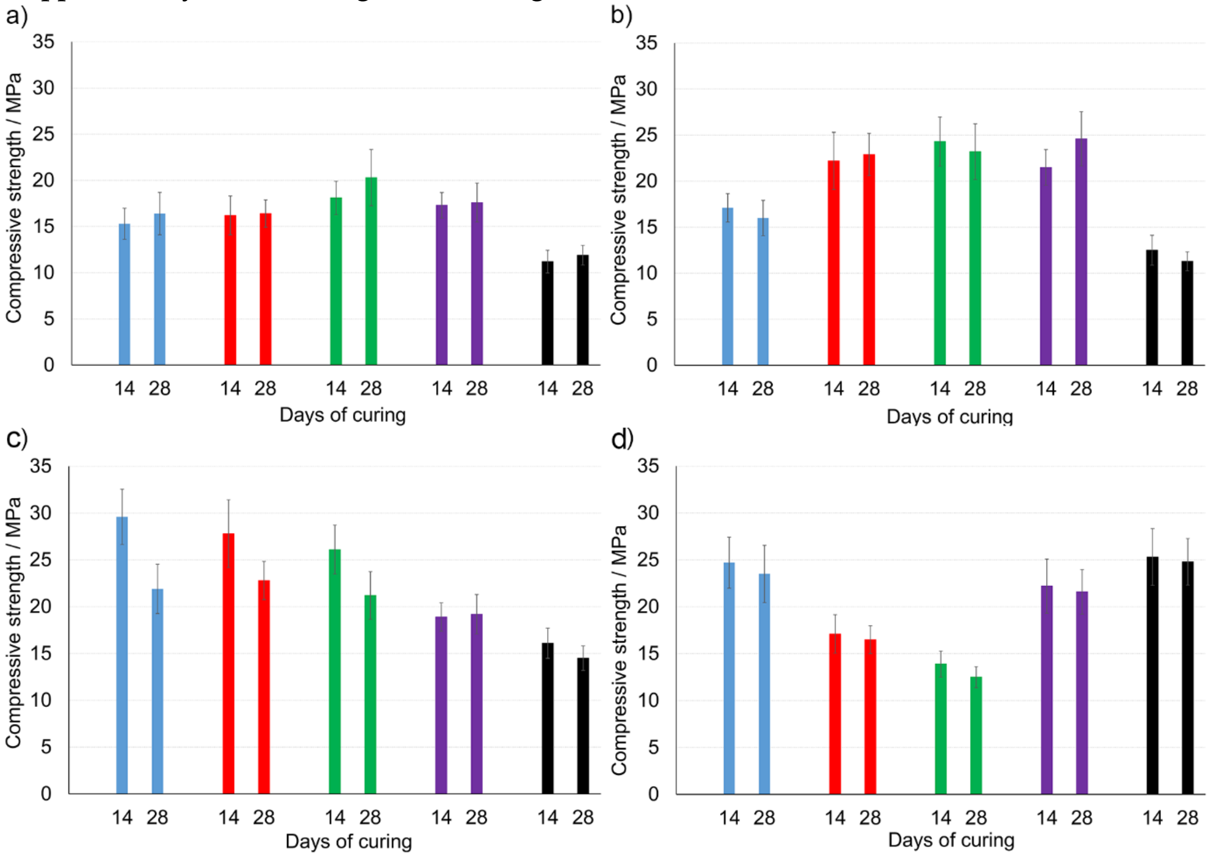

Figure S1. Compressive strength of the alkali-activated cement formed from post-production clay mixed with sand; calcined at two different temperatures: (a) 700 °C and (b) 750 °C; or formed from post-production clay mixed with sand and additives: (c) fly ash, (d) metakaolin; calcined at 750 °C; activated by NaOH (blue bars), NaOH:KOH 9:1 (red bars), NaOH:KOH 3:1 (green bars), NaOH:KOH 1:1 (purple bars), and KOH (black bars); cured for 14 and 28 days.

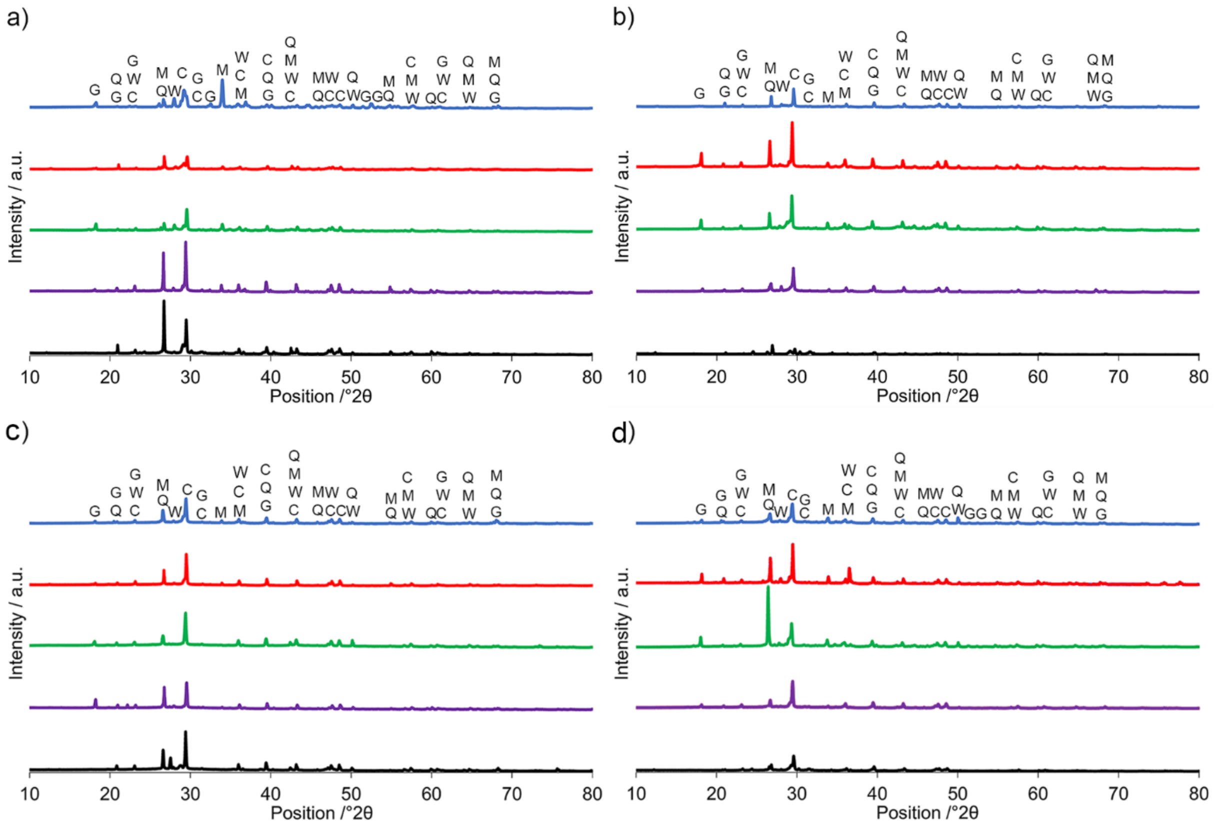

Figure S2. X-ray diffraction pattern of the alkali-activated cement formed from post-production clay mixed with sand; calcined at two different temperatures: (a) 700 °C and (b) 750 °C; or formed from post-production clay mixed with sand and additives: (c) fly ash, (d) metakaolin; calcined at 750 °C; activated by NaOH (blue), NaOH:KOH 9:1 (red), NaOH:KOH 3:1 (green), NaOH:KOH 1:1 (purple), and KOH (black); cured for 28 days.
